# Genomic prediction for yield and malting traits in barley using metabolomic and near-infrared spectra

**DOI:** 10.1007/s00122-024-04806-7

**Published:** 2025-01-09

**Authors:** Miguel A. Raffo, Pernille Sarup, Just Jensen, Xiangyu Guo, Jens D. Jensen, Jihad Orabi, Ahmed Jahoor, Ole F. Christensen

**Affiliations:** 1 Center for Quantitative Genetics and Genomics, Aarhus University, Aarhus C, Denmark; 2 Nordic Seed A/S, Odder, Denmark; 3 Danish Pig Research Centre, Danish Agriculture & Food Council, Copenhagen V, Denmark

## Abstract

**Key message:**

Genetic variation for malting quality as well as metabolomic and near-infrared features was identified. However, metabolomic and near-infrared features as additional omics-information did not improve accuracy of predicted breeding values.

**Abstract:**

Significant attention has recently been given to the potential benefits of metabolomics and near-infrared spectroscopy technologies for enhancing genetic evaluation in breeding programs. In this article, we used a commercial barley breeding population phenotyped for grain yield, grain protein content, and five malting quality traits: extract yield, wort viscosity, wort color, filtering speed, and β-glucan, and aimed to: (i) investigate genetic variation and heritability of metabolomic intensities and near-infrared wavelengths originating from leaf tissue and malted grain, respectively; (ii) investigate variance components and heritabilities for genomic models including metabolomics (GOBLUP-MI) or near-infrared wavelengths (GOBLUP-NIR); and (iii) evaluate the developed models for prediction of breeding values for traits of interest. In total, 639 barley lines were genotyped using an iSelect9K-Illumina barley chip and recorded with 30,468 metabolomic intensities and 141 near-infrared wavelengths. First, we found that a significant proportion of metabolomic intensities and near-infrared wavelengths had medium to high additive genetic variances and heritabilities. Second, we observed that both GOBLUP-MI and GOBLUP-NIR, increased the proportion of estimated genetic variance for grain yield, protein, malt extract, and β-glucan compared to a genomic model (GBLUP). Finally, we assessed these models to predict accurate breeding values in fivefold and leave-one-breeding-cycle-out cross-validations, and we generally observed a similar accuracy between GBLUP and GOBLUP-MI, and a worse accuracy for GOBLUP-NIR. Despite this trend, GOBLUP-MI and GOBLUP-NIR enhanced predictive ability compared to GBLUP by 4.6 and 2.4% for grain protein in leave-one-breeding-cycle-out and grain yield in fivefold cross-validations, respectively, but differences were not significant (*P-value* > 0.01).

**Supplementary Information:**

The online version contains supplementary material available at 10.1007/s00122-024-04806-7.

## Introduction

Barley (*Hordeum vulgare* L.) is a widely cultivated cereal crop primarily utilized for animal feed and malting for alcoholic beverage production (Miralles et al. 2021; Verma et al. 2022). Over the last decades, a growing demand for malting barley has increased the necessity for commercial varieties that integrate high productivity and superior malting quality. Grain yield (GY) and grain protein content (PC) are two of the most important traits in barley production. Developing varieties with high grain yield potential and protein content in the range of 9.0–11.5% are central goals in barley breeding programs (Bertholdsson 1999; Emebiri 2015; Barmeier et al. 2017). These traits can only be assessed late in the breeding process when there are enough seeds for replicated field trials. Malting quality refers to the capacity to undergo a successful malting process, which ultimately influences the flavor, aroma, and overall quality of the resulting beverage. Key traits influencing malting quality include malt extract yield ≥ 80%, malt protein between 9.5 and 12.5%, β-glucan from < 0.2 to 1.5%, soluble protein between 4 and 6%, diastatic power between 70 and120°L, wort viscosity between 1.5 and 5.0 cP, and low enzyme concentration (α-amylase, β-amylase, limit dextrinase, and β-glucosidase), among other traits (Li et al. 2009; Guo et al. 2020; Sarup et al. 2020). The assessment of malting quality is a complex and expensive process as it depends on numerous interrelated traits that collectively contribute to producing high-quality malt. Therefore, this assessment is usually not available for all breeding lines and replicate samples. Most importantly, just as the phenotypes for grain yield and protein content, reliable information of malting quality is not available in the early stages of the breeding cycle where selection is most intense. Breeding for the improvement of yield and malting quality traits in barley is challenged by assessments of these traits only being available late in the breeding process.

Genomic selection (GS, Meuwissen et al. 2001) based on whole-genome prediction (WGP) is a cost-effective methodology that can enhance accuracy in the early stages of selection, and it has been successfully utilized in plant and animal breeding to improve traits of economic importance (Crossa et al. 2010; Hayes and Goddard 2010; Raffo and Jensen 2023). To further extend and improve genomic selection breeding programs, there has been an increasing interest in exploiting omics technologies (Fakrudin et al. 2012; Chaudhary et al. 2019). The omics approaches generate a large quantity of data that can be seen as intermediate phenotypes (i.e., endophenotypes) between the DNA action and the final phenotype. Some examples of the utilization of omics in the context of genetic analysis and breeding can be found for metabolomics (Riedelsheimer et al. 2012; Hayes et al. 2017; Guo et al. 2022, 2023), transcriptomics (Guo et al. 2016; Delrot et al. 2020; Morgante et al. 2020), and proteomics (Zhu et al. 2021). In addition, other techniques measuring chemometric traits, such as near-infrared spectroscopy (NIRS), have been proposed (Hayes et al. 2017; Rincent et al. 2018; Robert et al. 2022). Similarly to genomics, different omics features, and near-infrared (NIR) wavelengths can be utilized to predict the phenotype and genetic values (Riedelsheimer et al. 2012; Hayes et al. 2017; Rincent et al. 2018; Christensen et al. 2021; Derbyshire et al. 2022; Robert et al. 2022).

In this article, we focus on the utilization of metabolomics and NIR data originating from nuclear magnetic resonance (NMR) spectroscopy and NIRS, respectively. The NMR spectroscopy is a powerful analytical technique that produces a high-dimensional set of signal intensities that can be associated with specific metabolites (Gunther et al. 1980); the signal intensities will be referred to as metabolomic intensities (MIs) hereinafter. The metabolomics data has been successfully utilized for the prediction of complex traits in maize (Riedelsheimer et al. 2012), rice (Xu et al. 2016), wheat (Hayes et al. 2017), barley (Guo et al. 2022), and other plant and animal species (reviewed by Fernandez et al. 2021; Scossa et al. 2021, and Sakurai 2022). The NIRS is a low-cost, non-destructive technique that quantifies absorbance/reflectance of biological samples at a broad range of wavelengths in the visible and NIR spectrum. The NIR wavelengths are routinely used in cereal breeding programs to predict water and protein content (Dowell et al. 2006; Osborne 2006) and are often available for any other purpose without additional costs. Recently, Rincent et al. (2018) proposed an alternative called phenomic selection, where NIR wavelengths are used for prediction of phenotypes. Phenomic selection has been successfully used to predict complex traits in wheat (Rincent et al. 2018; Cuevas et al. 2019; Krause et al. 2019; Robert et al. 2022), maize (Lane et al. 2020), rye (Galán et al. 2020), triticale (Zhu et al. 2021), Soybean (Parmley et al. 2019; Zhu et al. 2021), and poplar (Rincent et al. 2018).

Different methods have been proposed to incorporate omics or NIRS data in statistical genetic models. The MIs and NIR wavelengths can be included as regressors in genomic-like omics-based (GLOB) prediction models (Robert et al. 2022), where all variables can be directly incorporated as separated random effects or via similarity matrices (Riedelsheimer et al. 2012; Guo et al. 2016; Rincent et al. 2018; Schrag et al. 2018; Brault et al. 2022). However, while several approaches have provided insights into the relevance of MIs or NIR wavelengths on the trait, they do not generate predictions of omics- or NIR-based genetic effects that can be directly used for breeding purposes. For this purpose, Christensen et al. (2021) proposed to use a joint model that generates genomic estimated breeding values (GEBVs) as a combination of estimates of direct genomic effects and omics-mediated genomic effects (GOBLUP). This model has recently been implemented using genomics and metabolomics for barley (Guo et al. 2023) and microbiome data in sheep (Boggio et al. 2023), and the GOBLUP model can also be potentially useful to exploit NIR data.

In this study, we used a spring barley breeding population phenotyped for grain yield (GY), grain protein content (PC), and five malting quality traits: malt extract yield (EY), wort viscosity (WV), wort color (WC), filtering speed (FS), and β-glucan content (BG), and with MIs originating from NMR on leaf tissue and NIR wavelengths originating from whole grain after malting from the same experimental plots. We had three specific objectives:

(i) To investigate the genetic variation and heritability of MIs and NIR wavelengths.
(ii) To investigate variance components (VCs) and heritabilities for genetic models including genomic and metabolomics (GOBLUP-MI) or genomic and NIR wavelengths (GOBLUP-NIR) for all the available traits.
(iii) To evaluate the performance of the developed models (GBLUP, GOBLUP-MI and GOBLUP-NIR) for prediction of breeding values for the traits included.

The accuracies of predicted breeding values were evaluated using fivefold and leave-one-breeding-cycle-out (LBCO) cross-validation (CV) schemes, and results from both models were compared with a baseline genomic model (GBLUP).

## Materials and methods

### Experimental data

The plant material consisted of 639 sixth-generation (F_6_) spring barley (*Hordeum vulgare* L.) lines tested in 2,250 individual plots by the breeding company Nordic Seed A/S. The descriptive statistics for GY, PC, and the MQ traits WV, BG, EY, FS, and WC are presented in Table 1. The breeding lines came from two breeding cycles tested in years 2021 to 2022 in two locations in Denmark (DK): Odder (Central DK), Holeby (South-East DK) and Skive (North-West DK). A breeding cycle is defined as all crosses made within a single calendar year. The breeding lines within each year-location combination were arranged in field trials (i.e. experimental blocks) following a randomized incomplete block design. The field trials were divided into smaller plots of size 8.25 m^2^ (5.5 × 1.5 m), where in each plot a barley breeding line or a control line was sown. Two control barley lines were sown with three replications in each trial. The grain yield (kg/8.25 m^2^) and protein content (%) estimated by NIR spectra on raw grain using a PerkinElmer DA 7440 On-line NIR instrument were recorded on each plot. Grain samples from each plot were collected and processed in micro-malting batches to obtain several malting quality traits: malt extract yield (%), wort viscosity (mPa-s), wort color (European Brewery Convention units), filtering speed (cm/20 min), and β-glucan (mg/L). A detailed description of the methodology utilized to obtain malting quality traits can be found in Sarup et al. (2020).

**Table 1:** Descriptive statistics for grain yield, protein content, and malting quality traits

Trait^†^ (unit)	Minimum	Mean	Maximum	Standard deviation	Coefficient of variation (%)
GY (kg/8.25m^2^)	4.92	7.19	8.81	0.61	8.45
PC (%)	8.80	10.75	13.10	0.57	5.32
WV (mPa-s)	1.33	1.47	1.99	0.05	3.40
BG (mg/L)	70.00	153.00	730.00	3.71	76.60
EY (%)	76.76	82.28	95.50	1.44	1.74
FS (cm/20 min)	1.40	4.91	6.80	1.07	21.73
WC (EBC units)	2.40	5.11	7.31	0.53	10.33

^**†**^All traits were evaluated for 639 sixth-generation (F_6_) barley lines with 2,250 observations. GY: grain yield; PC: protein content; WV: wort viscosity; BG: β-glucan; EY: extract yield; FS: filtering speed; WC: wort color; EBC: European Brewery Convention units

The DNA extraction was performed using a modified CTAB method (Rogers and Bendich, 1985). The plant material was genotyped using an Illumina iSelect9K barley chip. A total of 8,198 single-nucleotide polymorphism (SNP) markers were utilized. Quality control was done by removing SNPs with minor allele frequency (MAF) lower than 5% and call rate lower than 0.90. Genotypes were coded 0,1,2, counting the number of alleles of the reference allele for each locus. Missing genotypes were ~ 0.3% and were assigned two times the observed allele frequency (i.e., mean dosage).

For each plot, the metabolomic information was obtained from 10 cuts of green flag leaf tips randomly distributed in the yield plot just after flag leaf appearance. All samples from the same location, year, and trial were collected in Eppendorph tubes on the same date within 3 h. The tubes were stored on dry ice in the field and subsequently frozen at -20 °C. The tissue samples were freeze dried and thereafter pulverized using a TissueLyser II (Qiagen®), after which 1.0 ml 50% methanol was added to the tubes. The samples were incubated in a Thermo shaker (TS-DW, Biosan) at 50 °C for 10 min and cooled to room temperature. After 5 min at 4000G in a centrifuge (4-5C, Sigma), 0.70 ml of supernatant was transferred to 2 ml Eppendorph tubes and frozen at − 20 °C until shipment to the NMR laboratory in one batch per year (3 months after harvest). The samples were shipped on dry ice and stored at − 80 °C upon arrival to the Swedish NMR center at the University of Gothenburg, Sweden. For NMR analysis, samples were put in CentriVap lyophilizer to dry for 2 h, setting at 20 °C. Following, 60 µl methanol-d4 was added to each tube and dried again for one hour. Then 600 µl PREC buffer was added to the samples (37.5 mM NaPi pD 6.95, 0.05% NaN3, 99.8% D2O, 0.747 mM TSP-d4). The samples were shaken at 800 rpm, 25 °C for 45 min and transferred to 5 mm SampleJet rack tubes. Acquisition was performed as IVDr 32-scan 1D 1H NOESY. In total, 30,468 metabolomic intensities (MIs) were recorded from one-dimensional (1D) 1H nuclear magnetic resonance (NMR) spectroscopy. The signal intensities were integrated over small chemical shift intervals along the spectra expressed in parts per million ranging from 0.70 to 9.00 ppm. For a full description of the procedure to prepare NMR and obtain MIs, see Guo et al. (2020).

The NIR data was obtained from whole grain after malted. A total of 141 NIR wavelengths were recorded for absorbance from 950 to 1650 nm with a step of 5 nm. The resulting wavelengths were treated according to Rincent et al. (2018) as follows: i) the NIR wavelengths were normalized (centered to zero and scaled to variance one), and ii) the first derivative was computed using a Savitzky-Golay filter (Savitzky and Golay 1964) implemented in the R package signal (Signal Developers 2014). The Savitzky-Golay first derivative is a preprocessing step used to reduce random noise produced by instrumental fluctuations and environmental interference, and for feature enhancement, preserving the important spectral features and amplifying changes in absorbance. See supplementary material 1 for an illustration of NIR wavelengths before and after Savitzky-Golay transformation. In the end, all 2,250 plots were characterized using MIs and NIR wavelengths.

### Estimation of heritability of MIs and NIR wavelengths

Two univariate models were utilized to estimate variance components (VCs) and narrow-sense heritabilities (${h}^{2}$) for MIs (Model-MI) and NIR wavelengths (Model-NIR). The Model 1 was defined as:

1 $$y_{j} = Xb_{j} + Z_{g} g_{j} + Z_{l} l_{j} + Z_{{i_{g} }} i_{gj} + Z_{{i_{l} }} i_{lj} + Z_{s} s_{j} + e_{j}$$

where ${{\varvec{y}}}_{j}$ is the vector of phenotypes for MIs ($j=1, \dots , \text{30,468}$); ${\varvec{X}}$ is the design matrix for the fixed effects; ${{\varvec{b}}}_{j}$ is the vector of fixed effects (year-location-trial); ${{\varvec{g}}}_{j}$ is the vector of genomic breeding values of the lines with ${{\varvec{g}}}_{j} \sim N(0,{\varvec{G}}{\sigma }_{{g}_{j}}^{2})$, where ${\sigma }_{{g}_{j}}^{2}$ is the additive genomic variance and ${\varvec{G}}$ is the genomic relationship matrix (VanRaden 2008): ${\varvec{G}}=\frac{{\varvec{Q}}{{\varvec{Q}}}^{\boldsymbol{^{\prime}}}}{2\sum {p}_{i}(1-{p}_{i})}$, with ${\varvec{Q}}$ being the genotypic matrix centered by two times the observed allele frequencies of the reference alleles, and ${p}_{i}$ is the allele frequency for the ${i}^{th} SNP$; ${{\varvec{l}}}_{j}$ is the vector of genetic line effects, which includes non-additive genetic effects, such as epistasis, and additive effects not explained by marker genotypes, with ${{\varvec{l}}}_{j} \sim N(0,{\varvec{I}}{\sigma }_{{l}_{j}}^{2})$, where ${\sigma }_{{l}_{j}}^{2}$ is the variance of line effects; ${{\varvec{i}}}_{gj}$ and ${{\varvec{i}}}_{lj}$ are vectors of genotype-by-environment interactions with the environment defined as the year-location combination, where $${{\varvec{i}}}_{gj} \sim N(0,\left[\begin{array}{c}\begin{array}{cc}{\varvec{G}}& 0\\ 0& {\varvec{G}}\end{array}\begin{array}{cc}\boldsymbol{ }\boldsymbol{ }\boldsymbol{ }\boldsymbol{ }0& 0\\ \boldsymbol{ }\boldsymbol{ }\boldsymbol{ }\boldsymbol{ }0& 0\end{array}\\ \begin{array}{cc}0& 0\\ 0& 0\end{array}\begin{array}{cc}\boldsymbol{ }\boldsymbol{ }\boldsymbol{ }\boldsymbol{ }{\varvec{G}}& 0\\ \boldsymbol{ }\boldsymbol{ }\boldsymbol{ }\boldsymbol{ }0& {\varvec{G}}\end{array}\end{array}\right]{\sigma }_{{i}_{gj}}^{2})$$ and ${{\varvec{i}}}_{lj} \sim N(0, {\varvec{I}}{\sigma }_{lj}^{2})$, with ${\sigma }_{gj}^{2}$ being the additive genomic-by-environment interaction variance, and ${\sigma }_{lj}^{2}$ being the genetic line-by-environment interaction variance due to additive genetic effects not accounted for by marker genotypes and non-additive genetic effects; ${{\varvec{s}}}_{j}$ is the vector of spatial effects, with ${{\varvec{s}}}_{j} \sim N(0,{\varvec{S}}{\sigma }_{{s}_{j}}^{2})$, where ${\varvec{S}}$ is a spatial similarity matrix computed as: ${\varvec{S}}=\frac{\mathbf{W}{\mathbf{W}}^{\mathbf{^{\prime}}}}{tr\left(\mathbf{W}{\mathbf{W}}^{\mathbf{^{\prime}}}\right)/n}$, where $\mathbf{W}$ is an indicator matrix relating the position of the target plot and their eight surrounding plots (neighboring plots) with the observations, $tr$ is the trace (sum of diagonal elements) and $n$ the total number of rows, and ${\sigma }_{{s}_{j}}^{2}$ is the variance of the ${{\varvec{s}}}_{j}$ effect (see Raffo et al. 2022 and Tessema et al. 2024 for a detailed description of this spatial effect), note that the degree of similarity in ${\varvec{S}}$ depend on the distance between plots; ${{\varvec{Z}}}_{g}$, ${{\varvec{Z}}}_{l}$, ${{\varvec{Z}}}_{{i}_{g}}$, ${{\varvec{Z}}}_{{i}_{l}}$, and ${{\varvec{Z}}}_{s}$ are the design matrices for ${{\varvec{g}}}_{j}$, ${{\varvec{l}}}_{j}$, ${{\varvec{i}}}_{gj}$, ${{\varvec{i}}}_{lj}$, and ${{\varvec{s}}}_{j}$, respectively; ${{\varvec{e}}}_{j}$ is a vector of random residual effect with ${{\varvec{e}}}_{j} \sim N(0,{\varvec{I}}{\sigma }_{{e}_{j}}^{2})$, where ${\sigma }_{{e}_{j}}^{2}$ is the residual variance.

Model 2 was defined for NIR wavelengths ($j=141$) with the same effects as in Model 1 plus an additional random effect ${{\varvec{Z}}}_{m}{{\varvec{m}}}_{j}$, corresponding to the batch in which the samples were malted, where ${{\varvec{m}}}_{j}$ is the vector of malting batch effects with ${{\varvec{m}}}_{j} \sim N(0,{\varvec{I}}{\sigma }_{{m}_{j}}^{2})$, and ${\sigma }_{{m}_{j}}^{2}$ is the variance of the malting batch effects; ${{\varvec{Z}}}_{m}$ is the design matrix for ${{\varvec{m}}}_{j}$. Note that Models 1 and 2 are adapted versions of GBLUP models (Habier et al. 2007; VanRaden 2008).

VCs were estimated by Restricted Maximum Likelihood (REML) using the Average Information (AI-REML) module in DMU (Madsen and Jensen, 2013). The heritability at the level of single-plot measurement was estimated for each MI and NIR wavelength as ${\widehat{h}}_{j}^{2}=d\left(\mathbf{G}\right){ \widehat{\sigma }}_{{g}_{j}}^{2}/{\widehat{\sigma }}_{{{\varvec{P}}}_{j}}^{2}$, where $d\left(\mathbf{G}\right)$ is the average of diagonal elements of the genomic relationship matrix $d\left(\mathbf{G}\right)=1.83$,${\widehat{\sigma }}_{{g}_{j}}^{2}$ is the estimated additive genomic variance, and ${\widehat{\sigma }}_{{P}_{j}}^{2}$ is the estimated phenotypic variance for each MI or NMR wavelength. The ${\widehat{\sigma }}_{{P}_{j}}^{2}$ for Model 1 was estimated as: ${\widehat{\sigma }}_{{P}_{j}}^{2}=$ $d\left(\mathbf{G}\right){ \widehat{\sigma }}_{{g}_{j}}^{2}+{\widehat{\sigma }}_{{l}_{j}}^{2}+d\left(\mathbf{G}\right){ \widehat{\sigma }}_{{i}_{gj}}^{2}+{\widehat{\sigma }}_{{i}_{lj}}^{2}+(d\left(\mathbf{S}\right)-mean\left(\mathbf{S}\right))* {\widehat{\sigma }}_{{s}_{j}}^{2}+{\widehat{\sigma }}_{{e}_{j}}^{2}$, where ${\widehat{\sigma }}_{{l}_{j}}^{2}$, ${\widehat{\sigma }}_{{i}_{gj}}^{2}$, ${\widehat{\sigma }}_{{i}_{lj}}^{2}$, ${\widehat{\sigma }}_{{s}_{j}}^{2}$, and ${\widehat{\sigma }}_{{e}_{j}}^{2}$ are the estimated parameters, $d\left(\mathbf{G}\right)$ is 1, and $mean\left(\mathbf{S}\right)$ is 0.004. The ${\widehat{\sigma }}_{{P}_{j}}^{2}$ for Model 2 was estimated using the same formula as Model 1 plus the variance of the malting batch effects ${\widehat{\sigma }}_{{m}_{j}}^{2}$. In addition, a significance test for ${\widehat{h}}_{j}^{2}$ equal to zero was performed using a simulation procedure to determine the null distribution of ${\widehat{h}}_{j}^{2}$ in our specific statistical design. This was done following the procedure proposed by Guo et al. (2020); see description in their article for further details.

### Genomic and multi-omics models for yield and malting traits

#### GBLUP

The GBLUP model (Habier et al. 2007; VanRaden 2008) was utilized for the traits GY, PC, EY, WV, WC, FS, and BG. For analyses of GY and PC, GBLUP was defined with the same model effects as described in the previous subsection for Model 1. For analyses of MQ traits EY, WV, WC, FS, and BG, GBLUP was defined as previously described for Model 2.

#### GOBLUP-MI

The GOBLUP model was proposed by Christensen et al. (2021) to integrate different omics data into genetic evaluations. In brief, the MBLUP is a joint model assuming that the phenotype of interest is affected by different omics expression levels (in addition to genomic effects), and where GEBVs can be obtained from a combination of genomic and omics information. See Christensen et al. (2021) for a complete theoretical description of the model and derivation of GEBVs. In our study, GOBLUP-MI refers to the model including MIs and GOBLUP-NIR to the model including NIR wavelengths. The model of Christensen et al. (2021) (GOBLUP-MI) for GY and PC was implemented using the following two steps:

step 1:

2 $$y = Xb_{1} + u + Z_{g} g_{1} + Z_{l} l_{1} + Z_{{i_{g} }} i_{g1} + Z_{{i_{l} }} i_{l1} + Z_{s} s_{1} + e_{1}$$

step 2:

3 $$\hat{u} = Xb_{2} + Z_{g} g_{2} + Z_{l} l_{2} + Z_{{i_{g} }} i_{g2} + Z_{{i_{l} }} i_{l2} + Z_{s} s_{2} + e_{2}$$

where ${\varvec{y}}$ is the vector of phenotypes, ${\varvec{X}}$, ${\varvec{b}}$, ${{\varvec{Z}}}_{g}$, ${{\varvec{Z}}}_{l}$, ${{\varvec{Z}}}_{{i}_{g}}$, ${{\varvec{Z}}}_{{i}_{l}}$, ${{\varvec{Z}}}_{s}$, ${\varvec{g}}$, ${\varvec{l}}$, ${{\varvec{i}}}_{g}$, ${{\varvec{i}}}_{l}$, ${\varvec{s}}$, ${\varvec{e}}$ are defined as for GBLUP, regardless of whether it is denoted with a subscript 1 or 2; ${\varvec{u}}$ is the vector of MIs effects on phenotype with ${\varvec{u}} \sim N(0,{{\varvec{Q}}}_{MI}{\sigma }_{{\varvec{u}}}^{2})$, where ${{\varvec{Q}}}_{MI}$ is the metabolomic similarity matrix computed as ${{\varvec{Q}}}_{MI}=\frac{{{\varvec{M}}}_{MI}{{\varvec{M}}}_{MI}^{\boldsymbol{^{\prime}}}}{q}$ with ${{\varvec{M}}}_{MI}$ a 2,250 (number of observations) × 30,468 (number of MIs) matrix of centered and scaled MIs, and ${\sigma }_{{\varvec{u}}}^{2}$ the metabolomic variance. In the step 2 (Eq. 3), $\widehat{{\varvec{u}}}$ is the vector of predicted metabolomic effect from step 1 (Eq. 2). The vector of GEBVs in GOBLUP-MI are therefore computed as the vector of GEBV in step 1 (${\widehat{{\varvec{g}}}}_{1}$) plus the vector of GEBVs in step 2 (${\widehat{{\varvec{g}}}}_{2}$). The GOBLUP-MI for MQ traits (EY, WV, WC, FS, and BG) was defined with the same effects as for GY and PC plus an additional random effect ${{\varvec{Z}}}_{m}{{\varvec{m}}}_{1}$ (for step 1) and ${{\varvec{Z}}}_{m}{{\varvec{m}}}_{2}$ (for step 2), corresponding to the mashing batch group in which samples were malted, where ${{\varvec{Z}}}_{m}$ and ${\varvec{m}}$ were defined as for GBLUP regardless of subscript 1 or 2.

#### GOBLUP-NIR

The GOBLUP-NIR was developed to include NIR wavelengths instead of MIs. This model had the same effects as GOBLUP-MI for all traits except for the metabolomic effects (${\varvec{u}}$), which was replaced by a new effect for NIR wavelengths. For step 1 of GOBLUP-NIR, the NIR effect was defined as ${\varvec{v}}$ (equivalent to ${\varvec{u}}$ in GOBLUP-MI), with ${\varvec{v}}$ as the vector of NIR wavelength effects on phenotype, where ${\varvec{v}} \sim N(0,{{\varvec{Q}}}_{NIR}{\sigma }_{{\varvec{v}}}^{2})$. To build the NIR relationship matrix ${{\varvec{Q}}}_{NIR}$, principal component analysis (PCA) was performed over the 141 centered and scaled NIR wavelengths for the complete population. The first eight principal components explained more than 99% of the variation and were utilized to compute ${{\varvec{Q}}}_{NIR}=\frac{{{\varvec{M}}}_{NIR}{{\varvec{M}}}_{NIR}^{\boldsymbol{^{\prime}}}}{t}$, where ${{\varvec{M}}}_{NIR}$ is a 2,250 (number of observations) × 8 (number of selected principal components) matrix, and ${\sigma }_{{\varvec{v}}}^{2}$ the estimated NIR wavelength variance. The principal components were used since it improved convergence of the REML algorithm used for VCs estimation compared to when NIR wavelengths were used directly. For step 2 of GOBLUP-NIR, the NIR wavelengths estimated effects were defined as $\widehat{{\varvec{v}}}$ (equivalent to $\widehat{{\varvec{u}}}$ in GOBLUP-MI). The GOBLUP-NIR was used for all traits except PC, as PC is directly predicted from NIRS. Note that in both models, GOBLUP-MI and GOBLUP-NIR, independence and equal heritabilities of all omics features are assumed.

### Variance and heritability estimation for GBLUP and GOBLUP for yield and malting quality traits

The VCs estimation was performed using the AI-REML module in the DMU software (Madsen and Jensen, 2013). For the GBLUP models, ${\widehat{h}}^{2}$ was computed using the same formulas as described for Models 1 and 2 in the section *"Estimation of heritability of MIs and NIR wavelengths"*; note that all these models have similar effects, but are used for different phenotypes (i.e., MIs, NIR wavelengths, GY, PC, or MQ traits).

The GOBLUP-MI allowed us to obtain different heritabilities than GBLUP. According to Christensen et al. (2021) the heritability in the GOBLUP can be defined as ${h}^{2}={h}_{d}^{2}+ {c}_{m}^{2}* {h}_{M}^{2}$; where ${h}_{d}^{2}$ is the direct heritability obtained from step 1 of MGLUP. For GOBLUP-MI, ${h}_{d}^{2}$ is estimated as ${\widehat{h}}_{d}^{2}=d\left(\mathbf{G}\right){ \widehat{\sigma }}_{{{\varvec{g}}}_{1}}^{2}/{ \widehat{\sigma }}_{{{\varvec{P}}}_{1}}^{2}$, with $d\left(\mathbf{G}\right)$ and ${\widehat{\sigma }}_{{{\varvec{g}}}_{1}}^{2}$ as previously defined, and ${\widehat{\sigma }}_{{{\varvec{P}}}_{1}}^{2}=d\left(\mathbf{G}\right){ \widehat{\sigma }}_{{{\varvec{g}}}_{1}}^{2}+d\left({{\varvec{Q}}}_{MI}\right){ \widehat{\sigma }}_{{\varvec{u}}}^{2}+ {\widehat{\sigma }}_{{{\varvec{l}}}_{1}}^{2}+d\left(\mathbf{G}\right){ \widehat{\sigma }}_{{{\varvec{i}}}_{{\varvec{g}}1}}^{2}+{\widehat{\sigma }}_{{{\varvec{i}}}_{{\varvec{l}}1}}^{2}+{(d\left(\mathbf{S}\right)-mean\left(\mathbf{S}\right))*\widehat{\sigma }}_{{{\varvec{s}}}_{1}}^{2}+{\widehat{\sigma }}_{{{\varvec{e}}}_{1}}^{2}$; ${c}_{m}^{2}$ is the metabolomics variance ratio, and is estimated as ${\widehat{c}}_{m}^{2}=d\left({{\varvec{Q}}}_{MI}\right){ \widehat{\sigma }}_{{\varvec{u}}}^{2}/{ \widehat{\sigma }}_{{{\varvec{P}}}_{1}}^{2}$; and ${h}_{M}^{2}$ is the heritability of MIs, and is estimated as ${\widehat{h}}_{M}^{2}=d\left(\mathbf{G}\right){ \widehat{\sigma }}_{{{\varvec{g}}}_{2}}^{2}/{ \widehat{\sigma }}_{{{\varvec{P}}}_{2}}^{2}$ with ${\widehat{\sigma }}_{{{\varvec{P}}}_{2}}^{2}=d\left(\mathbf{G}\right){ \widehat{\sigma }}_{{{\varvec{g}}}_{2}}^{2}+{\widehat{\sigma }}_{{{\varvec{l}}}_{2}}^{2}+d\left(\mathbf{G}\right){ \widehat{\sigma }}_{{{\varvec{i}}}_{{\varvec{g}}2}}^{2}+{\widehat{\sigma }}_{{{\varvec{i}}}_{{\varvec{g}}2}}^{2}+{(d\left(\mathbf{S}\right)-mean\left(\mathbf{S}\right))*\widehat{\sigma }}_{{{\varvec{s}}}_{2}}^{2}+{\widehat{\sigma }}_{{{\varvec{e}}}_{2}}^{2}.$ For GOBLUP-NIR, parameters were calculated using the same formulas as for GOBLUP-MI, but replacing ${{\varvec{Q}}}_{MI}$ with ${{\varvec{Q}}}_{NIR}$ for computing ${\widehat{\sigma }}_{{{\varvec{P}}}_{1}}^{2}$ and ${\widehat{c}}_{m}^{2}$ (NIR wavelength variance ratio in GOBLUP-NIR); the ${h}_{M}^{2}$ in GOBLUP-NIR represents the heritability of NIR wavelengths.

### Cross-validation and model validation

Predictions of GEBVs from GBLUP, GOBLUP-MI and GOBLUP-NIR were assessed using fivefold and leave-one-breeding-cycle-out (LBCO) CVs schemes. The fivefold CV consisted of randomly masking the phenotypes of all replicates of 20% of the lines and using the remaining lines to predict the additive genetic values. This process was repeated five times until all lines were assigned to one of the five folds and predicted. The fivefold CV is useful for predictions with high genetic relationship between reference population (RP) and validation population (VP) as relatives such as parents, siblings, and half-siblings can be shared between RP and VP. Thus, the fivefold allows us to investigate the performance of the genetic models in a scenario where a new variety is predicted via genotype but no phenotypic records is available. The LBCO CV was performed by masking the phenotypes of one breeding cycle in the VP and using the remaining phenotyped lines to predict the masked lines. This process was repeated twice to predict the breeding cycles evaluated in 2021 and 2022. The LBCO CV allowed us to evaluate the prediction problem where newly developed lines are predicted from parental and historical records. For GOBLUP-MI and GOBLUP-NIR both CVs were performed by masking both phenotypes and MIs (or NIR) information in the VP. The reason for masking MIs (or NIR) in VP is that environmental correlations may influence predictions if MIs (or NIR) wavelengths are not masked, and this would represent a similar scenario to the situation of a bivariate model with records for the secondary trait in VP (see Guo et al. 2023 for details). The models' predictive ability (PA) was evaluated as the correlation between GEBVs and phenotypes corrected by fixed effects (${{\varvec{y}}}_{c}$). In addition, the regression coefficient of GEBVs obtained with whole phenotypic information on GEBVs obtained with partial phenotypic information was used as an estimate of variance inflation: ${{\varvec{b}}}_{w,p}=\frac{cov({\widehat{g}}_{w}, {\widehat{g}}_{p})}{var({\widehat{g}}_{p})}$ (Legarra and Reverter 2018). The standard errors for PA and ${{\varvec{b}}}_{w,p}$ were obtained using an ordinary non-parametric bootstrapping with replacement, full sample size, and 10,000 replications. The PA between models for each trait was contrasted using a two-tailed paired *t*-test (critical *P-value* = 0.01).

### Ratios of accuracies

**Fig. 1:**
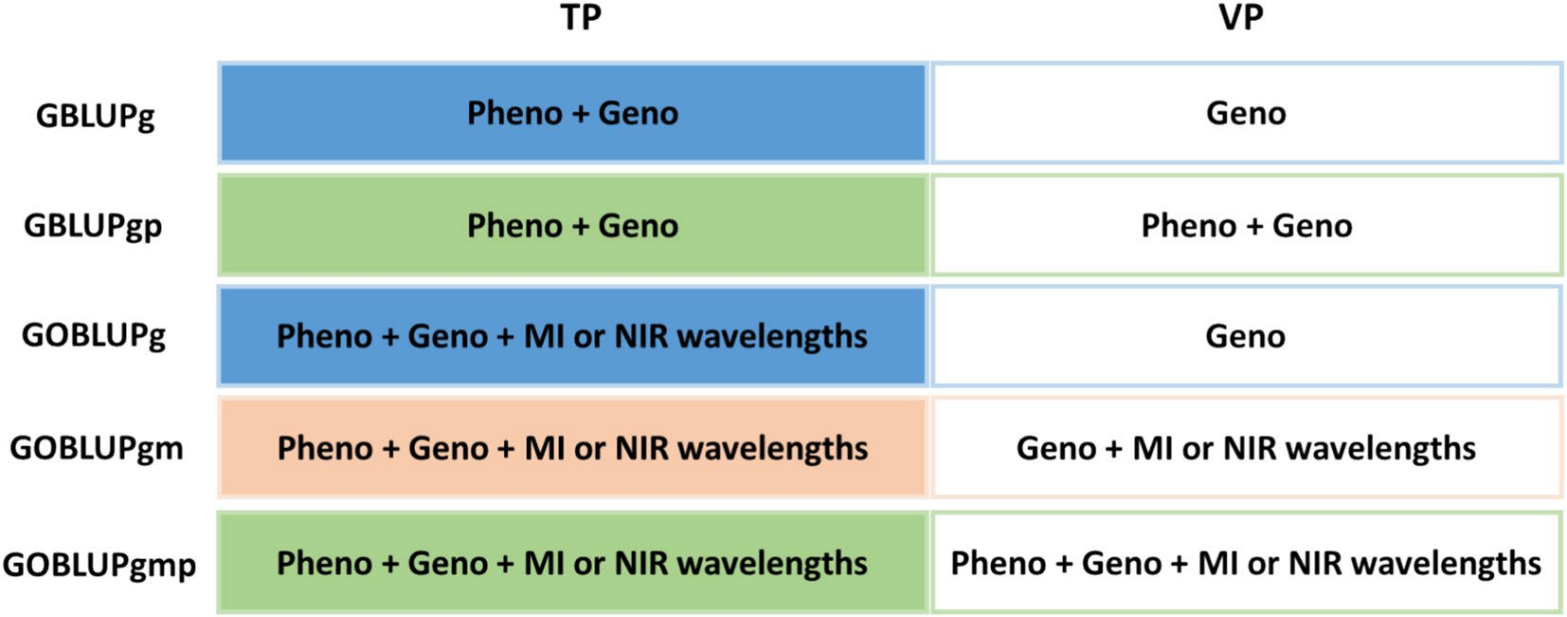
Allocation of training and validation sets for ratios of population accuracies analysis. TP: training population; VP: validation population; GBLUPg: GBLUP with genomic information in validation population; GBLUPgp: GBLUP with genomic and phenotypic information in validation population; GOBLUPg: GOBLUP with genomic information in validation population; GOBLUPgm: GOBLUP with genomic and metabolomic (or NIR) information in validation population; GOBLUPgmp: GOBLUP with genomic, metabolomic (or NIR) and phenotypic information in validation population; Pheno: phenotypic information, Geno: genomic information; MI: metabolomic intensities; NIR near-infrared

Different scenarios comparing ratios of population accuracies of predicted breeding values were assessed according to Lagarra and Reverter (2018). The ratios of accuracies are a measure of the increase in accuracy when including additional information to the models and can be estimated as the correlation between breeding values estimated with whole (${\widehat{{\varvec{g}}}}_{w}$) and partial information (${\widehat{{\varvec{g}}}}_{p}$); the lower the correlation, the larger the increase in accuracy by adding new information. Different ratios of accuracies were computed and named according to the different information in VP (Fig. 1). The ratios of accuracies computed were: i) GBLUPg/gp, representing the correlation of GEBV with genomic information in VP and GEBV with genomic and phenotypic information in VP, ii) GOBLUPg/gm as the correlation of GEBV with genomic in VP and GEBV with genomic and omics information in VP, iii) GOBLUPgm/gmp as the correlation of GEBV with genomic and omics in VP and GEBV with genomic, omics, and phenotypic information in VP, and iv) GOBLUPg/gmp as the correlation of GEBV with genomic in VP and GEBV with genomic, omics, and phenotypic information in VP.

## Results

### Estimates of heritability of metabolomic intensities and NIR wavelengths

**Fig. 2:**
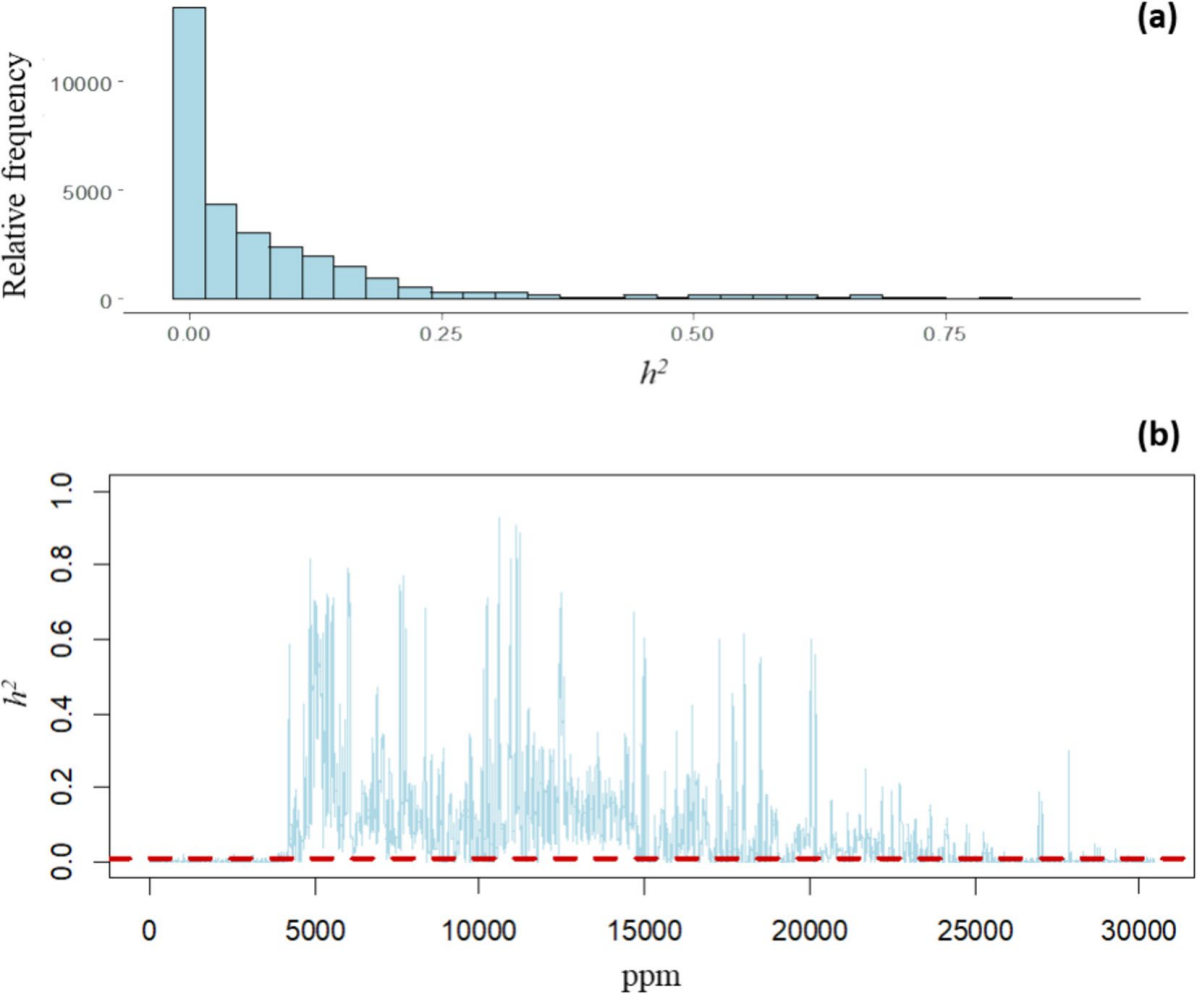
Estimated heritabilities for metabolomic intensities of barley leaf tissue; (**a**) histogram of estimated narrow-sense heritability (${\widehat{h}}^{2}$); (**b**) estimated narrow-sense heritability for the 30,468 metabolomic intensities (MIs) ordered by chemical shift in ppm, the horizontal red-dashed line is the significance level at ${\widehat{h}}^{2}$ of 0.0123 (significant threshold at 0.01)

**Fig. 3:**
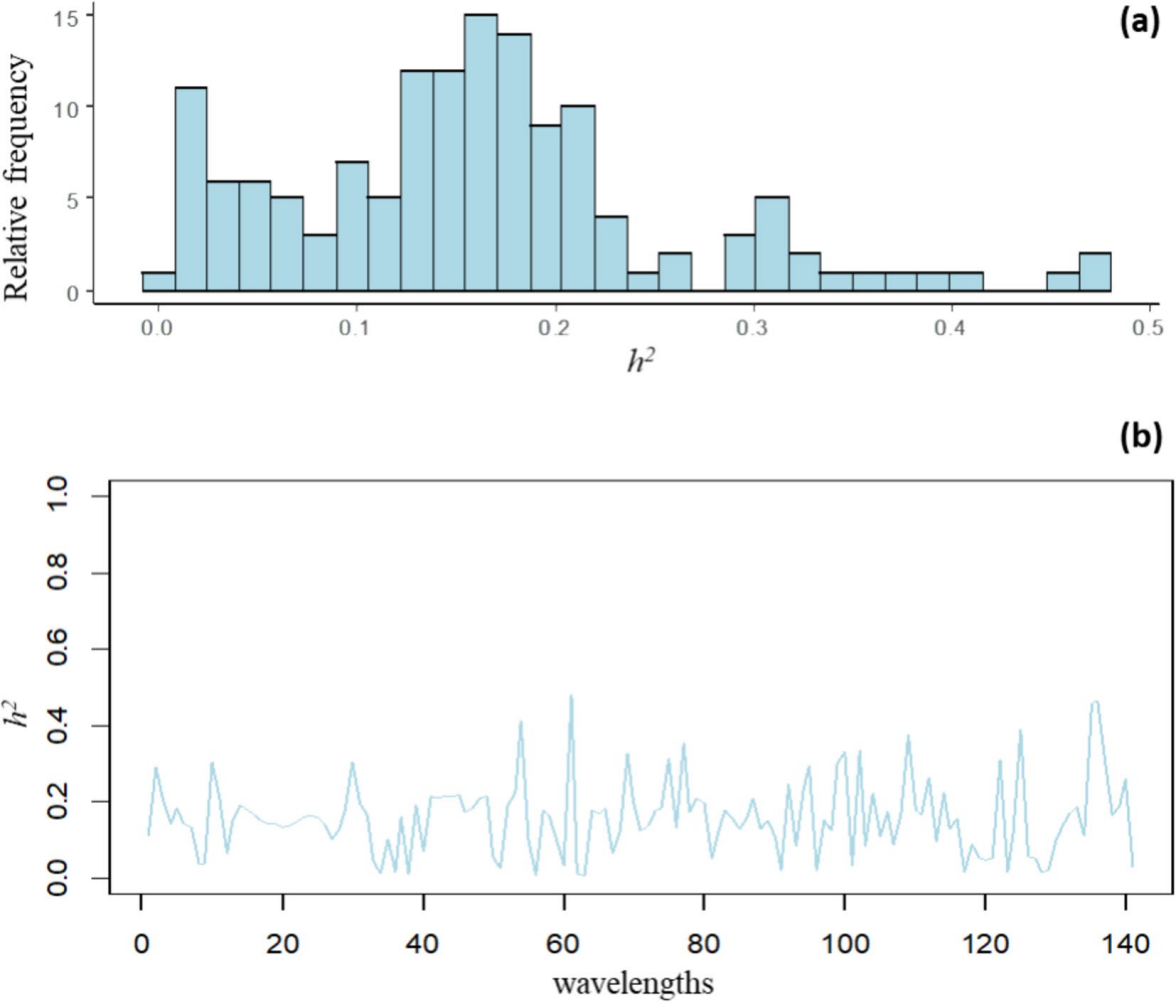
Estimated heritabilities for NIR wavelengths of barley on whole grain after malting for Savitzky-Golay transformation; (**a**) histogram of estimated narrow-sense heritability (${\widehat{h}}^{2}$); (**b**) estimated narrow-sense heritability for the 141 NIR wavelengths ordered by absorbance

The estimates of ${h}^{2}$ for each MI and each NIR wavelength are shown in Figs. 2 and 3, respectively. For the other VCs, the relative proportions of VCs for each MIs and NIR wavelengths are shown in supplementary material 2. The distribution of ${\widehat{h}}^{2}$ of MIs is displayed in the histogram in Fig. 2a. A wide range of ${\widehat{h}}^{2}$ was observed, with 40.22% of values lower than 0.01 and a maximum value of 0.93. The average ${\widehat{h}}^{2}$ of MIs was 0.08, with a median of 0.03 and a third quartile of 0.11. The ${\widehat{h}}^{2}$ for each of the 30,468 MIs ordered by chemical shift is shown in Fig. 2b. Across the different regions of the chemical shift interval, a trend of ${\widehat{h}}^{2}$ close to zero was seen for MIs at the beginning and end of the spectra, indicating that no biological signals were detected in these regions, 6.77% of MIs had moderate ${\widehat{h}}^{2}$ from 0.2 to 0.5, and 2.91% of Mis had ${\widehat{h}}^{2}$ larger than 0.5. The significance test for ${\widehat{h}}^{2}$ of MIs based on determination of the null distribution of ${\widehat{h}}^{2}$, resulted in a significance level at ${\widehat{h}}^{2}$ of 0.0123 estimated for a significance threshold of 0.01 (horizontal red-dashed line in Fig. 2b). A total of 17,677 out of 30,468 MIs (58.02%) significantly differed from zero in this statistical test.

The distribution of ${\widehat{h}}^{2}$ of NIR wavelengths is displayed in the histogram in Fig. 3a. This shows that 1.42% of ${\widehat{h}}^{2}$ were lower than 0.01 and a maximum value of 0.48 was observed. The average and median ${\widehat{h}}^{2}$ of NIR wavelengths were both 0.16, with a third quartile of 0.20. The ${\widehat{h}}^{2}$ for each of the 141 NIR wavelengths ordered by absorbance is shown in Fig. 3b. Across the different regions of the NIR spectra, 25.53% of wavelengths had moderate ${\widehat{h}}^{2}$ (> 0.2). In comparison with MIs, the NIR wavelengths had a lower proportion of very low or high ${\widehat{h}}^{2}$, and most of them were in the intermediate range.

### Variance components and heritabilities using GBLUP and GOBLUP models for yield and malting quality traits

The GBLUP, GOBLUP-MI and GOBLUP-NIR models were utilized to estimate VCs and population parameters for GY, PC (except for GOBLUP-NIR), and MQ traits.

The estimates of genetic (${\widehat{{\varvec{\sigma}}}}_{{\varvec{g}}}^{2}$) and phenotypic (${\widehat{{\varvec{\sigma}}}}_{{\varvec{P}}}^{2}$) variances are shown in Table 2. The other VCs and the standard deviations of estimates are shown in supplementary material 3. The VCs were successfully estimated with GBLUP and GOBLUP-MI for all traits. The GOBLUP-NIR was successfully utilized to estimate VCs for GY, WV, BG, and EY, but it was not possible for FS and WC due to the estimated NIR variance (${\widehat{\sigma }}_{v}^{2}$) converging towards zero. The ${\widehat{{\varvec{\sigma}}}}_{{\varvec{g}}}^{2}$ for GBLUP was higher than the direct genomic variance of GOBLUP-MI (${\widehat{{\varvec{\sigma}}}}_{{\varvec{g}}}^{2}$ in step 1) for GY and PC. This trend was not observed for MQ traits, where in general the ${\widehat{{\varvec{\sigma}}}}_{{\varvec{g}}}^{2}$ for GBLUP and the direct genomic variance of GOBLUP-MI were similar. The direct genomic variance of GOBLUP-NIR was slightly higher than that for GBLUP and GOBLUP-MI for GY, and it was slightly lower for the MQ traits. For each trait, the ${\widehat{{\varvec{\sigma}}}}_{{\varvec{P}}}^{2}$ differed between models, with the largest differences observed for BG and EY, where ${\widehat{{\varvec{\sigma}}}}_{{\varvec{P}}}^{2}$ for GOBLUP-NIR was considerably larger than for the other models. The ${\widehat{{\varvec{\sigma}}}}_{{\varvec{P}}}^{2}$ for BG and EY in the GOBLUP-NIR were higher than the raw phenotypic variance of the trait (Table 1). The ${\widehat{{\varvec{\sigma}}}}_{{\varvec{P}}}^{2}$ for GBLUP and GOBLUP-MI were in general in a similar range for GY, PC, WV, and WC, but it was higher for the GBLUP for BG and FS, and higher for the GOBLUP-MI for EY.

**Table 2:** Genetic and phenotypic variance estimates from GBLUP and GOBLUPs models

Trait	GBLUP		GOBLUP-MI				GOBLUP-NIR			
			step 1		step 2		step 1		step 2	
	$d\left(\text{G}\right){\widehat{\sigma }}_{g}^{2}$	${\widehat{\sigma }}_{P}^{2}$	$d\left(\text{G}\right){\widehat{\sigma }}_{g}^{2}$	${\widehat{\sigma }}_{P}^{2}$	$d\left(\text{G}\right){\widehat{\sigma }}_{g}^{2}$	${\widehat{\sigma }}_{P}^{2}$	$d\left(\text{G}\right){\widehat{\sigma }}_{g}^{2}$	${\widehat{\sigma }}_{P}^{2}$	$d\left(\text{G}\right){\widehat{\sigma }}_{g}^{2}$	${\widehat{\sigma }}_{P}^{2}$
GY	0.015	0.134	0.014	0.122	0.002	0.014	0.017	0.143	0.0006	0.017
PC	0.016	0.198	0.012	0.193	0.002	0.022	-	-	-	-
WV	3.94 E-04	0.002	3.94 E-04	1.80 E-03	3.05 E-07	1.92 E-06	2.26 E-04	7.44 E-03	3.87 E-04	1.77 E-03
BG	1817	11,831	1818	10,136	0.03	0.24	1162	23,062	579	1312
EY	0.095	0.685	0.099	0.873	0.001	0.010	0.094	5.161	0.027	0.099
FS	0.018	1.073	0.018	0.805	1.33 E-06	9.72 E-06	0.017	0.803	-	-
WC	0.092	0.244	0.091	0.239	9.07 E-06	0.0002	-	-	-	-

GY: grain yield; PC: protein content; WV: wort viscosity; BG: β-glucan; EY: extract yield; FS: filtering speed; WC: wort color; EBC: European Brewery Convention units; ${\varvec{d}}\left(\mathbf{G}\right){\widehat{{\varvec{\sigma}}}}_{{\varvec{g}}}^{2}$: estimated additive variance; ${\widehat{{\varvec{\sigma}}}}_{{\varvec{P}}}^{2}$: estimated phenotypic variance. Incomplete variances are presented for FS and WC in GOBLUP-due to NIR variance converged towards zero in step 1. Standard errors of estimates are presented in supplementary material 3

The estimates of genetic parameters and heritabilities for GBLUP, GOBLUP-MI, and GOBLUP-NIR models are shown in Table 3. The heritabilities varied depending on the model used; the highest values were obtained for WC (0.38–0.38), followed by WV (0.21–0.22), BG (0.18–0.33), EY (0.11–0.26), GY (0.11–0.13), PC (0.08), and the lowest was obtained for FS (0.02). Note that the ${\widehat{h}}^{2}$ estimated from GOBLUP models is the sum of a direct heritability (${\widehat{h}}_{d}^{2}$) and an omics-mediated heritability equal to the product of ${\widehat{c}}_{m}^{2}$ and ${\widehat{h}}_{M}^{2}$ (decomposed in Table 3). The proportion of ${\widehat{c}}_{m}^{2}$ for GOBLUP-MI was higher for GY (20.5%) and PC (25.5%) than for MQ traits (< 4%). The proportion of ${\widehat{c}}_{m}^{2}$ for GOBLUP-NIR was in a similar range to GOBLUP-MI for GY (22.3%), but it was considerably higher for WV (81.2%), BG (63.2%), and EY (85.3%). The ${\widehat{h}}_{M}^{2}$ for GOBLUP-MI was higher for WV (0.16) followed by FS (0.14), BG (0.14), GY (0.14), EY (0.11), and WC (0.04). The ${\widehat{h}}_{M}^{2}$ for GOBLUP-NIR was higher for BG (0.44), EY (0.28), WV (0.22), and GY (0.03). Comparing the two GOBLUP models, the GOBLUP-MI presented higher ${\widehat{h}}^{2}$ for GY and WV, but the GOBLUP-NIR presented higher ${\widehat{h}}^{2}$ for BG and EY, and both models presented from similar to higher ${\widehat{h}}^{2}$ than GBLUP for the different traits.

**Table 3:** Genetic parameters and heritability estimates from GBLUP and GOBLUPs models

Trait	GBLUP	GOBLUP-MI				GOBLUP-NIR			
	${\widehat{h}}^{2}$	${\widehat{h}}_{d}^{2}$	${\widehat{c}}_{m}^{2}$	${\widehat{h}}_{M}^{2}$	${\widehat{h}}^{2}$	${\widehat{h}}_{d}^{2}$	${\widehat{c}}_{m}^{2}$	${\widehat{h}}_{M}^{2}$	${\widehat{h}}^{2}$
GY	0.11	0.11	0.21	0.14	0.14	0.12	0.22	0.03	0.13
PC	0.08	0.06	0.26	0.09	0.08	-	-	-	-
WV	0.22	0.22	0.01	0.16	0.22	0.03	0.81	0.22	0.21
BG	0.18	0.18	< 0.01	0.14	0.18	0.05	0.63	0.44	0.33
EY	0.11	0.11	0.04	0.11	0.12	0.02	0.85	0.28	0.26
FS	0.02	0.02	< 0.01	0.14	0.02	0.02	< 0.01	-	-
WC	0.38	0.38	0.01	0.04	0.38	-	-	-	-

GY: grain yield; PC: protein content; WV: wort viscosity; BG: β-glucan; EY: extract yield; FS: filtering speed; WC: wort color; ${h}_{d}^{2}$: direct heritability; ${c}_{m}^{2}$: metabolomics (GOBLUP-MI) or NIR wavelengths (GOBLUP-NIR) variance ratio; ${h}_{M}^{2}$: heritability of MIs (GOBLUP-MI) or NIR wavelengths (GOBLUP-NIR). Bold numbers are narrow-sense heritability (${{\varvec{h}}}^{2}$) for each trais and model

### Prediction of breeding values

The PA and ${{\varvec{b}}}_{w,p}$ for GBLUP, GOBLUP-MI and GOBLUP-NIR models were investigated using fivefold (Table 4) and LBCO (Table 5) CV schemes.

**Table 4:** Predictive ability (PA) and slope of regression of estimated genetic values with whole information on genetic values with partial information (${{\varvec{b}}}_{w,p}$) for models in fivefold cross-validation

Trait	GBLUP		GOBLUP-MI		GOBLUP-NIR	
	PA | SE	${b}_{w,p}$	PA | SE	${b}_{w,p}$	PA | SE	${b}_{w,p}$
GY	0.41 (a)^†^| 0.04	1.00 (0.02)	0.39 (a) | 0.04	1.01 (0.02)	0.42 (a) | 0.04	1.01 (0.02)
PC	0.37 (a) | 0.03	1.00 (0.02)	0.37 (a) | 0.03	1.01 (0.02)	-	-
WV	0.48 (ab) | 0.03	1.00 (0.03)	0.48 (a) | 0.03	1.00 (0.03)	0.37 (b) |0.03	1.10 (0.03)
BG	0.32 (b) | 0.04	1.00 (0.03)	0.32 (a) | 0.04	1.00 (0.03)	0.27 (c) | 0.04	1.07 (0.04)
EY	0.32 (a) | 0.04	1.00 (0.02)	0.32 (a) | 0.04	0.99 (0.02)	0.27 (b) | 0.04	1.00 (0.02)
FS	0.23 (a) | 0.04	1.03 (0.01)	0.23 (a) | 0.04	1.03 (0.01)	-	-
WC	0.70 (a) | 0.02	1.00 (0.01)	0.70 (a) | 0.02	1.00 (0.01)	-	-

GY: grain yield; PC: protein content; WV: wort viscosity; BG: β-glucan; EY: extract yield; FS: filtering speed; WC: wort color. ^†^Differences in the letter in parentheses represent significant differences between models for each trait (*P-value* < 0.01). SE: bootstrap-based standard errors of estimates

**Table 5:** Predictive ability (PA) and slope of regression of estimated genetic values with whole information on genetic values with partial information (${{\varvec{b}}}_{w,p}$) for models in fivefold cross-validation

Trait	GBLUP		GOBLUP-MI		GOBLUP-NIR	
	PA | SE	${b}_{w,p}$	PA | SE	${b}_{w,p}$	PA | SE	${b}_{w,p}$
GY	0.13 (a)^†^ | 0.04	0.75 (0.04)	0.10 (b) | 0.04	0.68 (0.04)	0.13 (ab) | 0.04	0.68 (0.04)
PC	0.22 (a) | 0.04	0.97 (0.04)	0.23 (a) | 0.04	0.97 (0.04)	-	-
WV	0.43 (ab) | 0.05	0.99 (0.03)	0.43 (a) | 0.04	0.99 (0.03)	0.33 (b) | 0.05	1.07 (0.06)
BG	0.29 (a) | 0.06	0.92 (0.03)	0.29 (a) | 0.06	0.92 (0.03)	0.25 (a) | 0.06	0.96 (0.04)
EY	0.27 (a) | 0.04	1.02 (0.03)	0.26 (a) | 0.04	1.00 (0.03)	0.26 (a) | 0.04	0.88 (0.03)
FS	0.10 (a) | 0.04	0.90 (0.03)	0.10 (a) | 0.04	0.86 (0.03)	-	-
WC	0.60 (a) | 0.03	0.96 (0.02)	0.60 (a) | 0.03	0.94 (0.02)	-	-

GY: grain yield; PC: protein content; WV: wort viscosity; BG: β-glucan; EY: extract yield; FS: filtering speed; WC: wort color. ^†^Differences in the letter in parentheses represent significant differences between models for each trait (*P-value* < 0.01). SE: bootstrap-based standard errors of estimates

In fivefold CV (Table 4), the highest PA was observed for WC (0.70), followed by WV (0.37 to 0.48), GY (0.39 to 0.42), PC (0.37), BG (0.27 to 0.32), EY (0.27 to 0.32), and the lowest for FS (0.23). As a general trend, the PA between GBLUP and GOBLUP-MI was similar, and it was significantly higher for several traits than the PA for GOBLUP-NIR. For PC, the highest PA was obtained for GOBLUP-MI and it was 1.4% higher than for GBLUP (not significant at a critical *P-value* of 0.01). For WV, the highest PA was obtained for GBLUP and GOBLUP-MI, which was significantly higher (~ 30%) than for GOBLUP-NIR. A similar trend as in WV was observed for BG and EY, with significantly higher PA for GBLUP and GOBLUP-MI (~ 17%) compared to GOBLUP-NIR. However, for GY the highest PA was obtained using the GOBLUP-NIR model, followed by the GBLUP and GOBLUP-MI models. Here, the increase in PA provided by GOBLUP-NIR was low, representing a 2.4% increase compared to GBLUP and 8.3% compared to GOBLUP-MI; the differences for GY were not significant in the *t*-test (critical *P-value* of 0.01). No relevant differences in PA were observed between models for BG, FS, and WC. In the fivefold CV, the ${{\varvec{b}}}_{w,p}$ did not signal any significant under- or -over dispersion since values were around 1 for all models (Table 4).

In LBCO CV (Table 5), PAs were lower than in the fivefold CV. The highest PA was observed for WC (0.60), followed by WV (0.33 to 0.43), BG (0.25 to 0.29), EY (0.26 to 0.27), PC (0.22), GY (0.10 to 0.13), and FS (0.10). Similarly to fivefold CV, in LBCO there was a general trend of similar PA between GBLUP and GOBLUP-MI, and both models significantly outperformed the PA of GOBLUP-NIR for several traits. For GY, the highest PA was obtained using the GBLUP and GOBLUP-NIR models, which was ~ 39% higher than for GOBLUP-MI; these differences were significant (*P-value* < 0.01) between GBLUP and GOBLUP-MI but not between GOBLUP-NIR and GOBLUP-MI. For PC, the highest PA was obtained for GOBLUP-MI and represented a no significant increase of 4.6% compared to GBLUP. For WV, the highest PA was obtained using the GBLUP and GOBLUP-MI models and it was ~ 33% higher than for GOBLUP-NIR; these differences were significant (*P-value* < 0.01) between GOBLUP-MI and GOBLUP-NIR but not between GBLUP and GOBLUP-NIR. A similar trend as in WV was observed for BG, with the highest PA for GBLUP and GOBLUP-MI and an increase of 14.6% compared to GOBLUP-NIR, but differences were not significant between models. No relevant differences in PA were observed between models for BG, FS, and WC. The estimate for ${{\varvec{b}}}_{w,p}$ in LBCO differed depending on the trait and model. No significant under- or -over dispersion was observed for most traits; but some over-dispersion was observed for GY.

The ratios of population accuracies of predicted breeding values obtained with the LR method for fivefold and LBCO CV are presented in Tables 6 and 7, respectively. Ratios of accuracies close to one reveal that no relevant improvement by including new information for prediction is obtained. We observed that the ratios for fivefold CV (0.85 to 1.0) were higher than for the LBCO CV (0.61 to 1.0) for all traits. In fivefold CV, a moderate improvement was observed for including phenotypic information in VP for all models and traits (ratios from 0.87 to 0.95 in GBLUPgm/gmp and GOBLUP_s_gm/gmp). A similar trend but with a larger effect of including phenotypes in VP was observed in LBCO CV (ratios from 0.62 to 0.86 in GBLUPgm/gmp and GOBLUP_s_gm/gmp). The impact of including omics in VP can be observed by looking at the GOBLUPg/gm ratio. From GOBLUPg/gm, it can be observed that there was no relevant effect of including MI in any of the two CVs for GOBLUP-MI (GOBLUP-MIg/gm ratio ~ 1 for all traits). Similar results were observed for the GOBLUP-NIRg/gm ratio for GY in both CVs. The GOBLUP-NIRg/gm ratio for WV, BG and EY were about 0.95 for GOBLUP-NIR in fivefold and 0.90 for LBCO. In principle, it may suggest an improvement by using NIR wavelengths in GOBLUP-NIR, but considering the lower PA of GOBLUP-NIR for these traits, this improvement does not seem to represent an extra benefit of using NIR wavelengths in the predictive performance compared to the baseline GBLUP or GOBLUP-MI models.

**Table 6:** Ratio of population accuracies of predicted breeding values for GBLUP, GOBLUP-MI, and GOBLUP-NIR for fivefold cross-validation

Trait	GBLUPg/gp	GOBLUP-MI			GOBLUP-NIR		
		g/gm	gm/gmp	g/gmp	g/gm	gm/gmp	g/gmp
GY	0,90	0,99	0,91	0,90	1,00	0,90	0,90
PC	0,92	0,99	0,92	0,92	-	-	-
WV	0,88	1,00	0,88	0,88	0,94	0,94	0,87
BG	0,87	1,00	0,87	0,87	0,94	0,92	0,85
EY	0,90	1,00	0,90	0,90	0,96	0,91	0,88
FS	0,95	1,00	0,95	0,95	-	-	-
WC	0,95	1,00	0,95	0,95	-	-	-

GY: grain yield; PC: protein content; WV: wort viscosity; BG: β-glucan; EY: extract yield; FS: filtering speed; WC: wort color. GOBLUP_s_g/gm: ratio of accuracies for validation populations with genomic vs. genomic + phenotypes; GOBLUP_s_gm/gmp: ratio of accuracies for validation populations with genomic + omics vs. genomic + omics + phenotypes; GOBLUP_s_g/gmp: ratio of accuracies for validation populations with genomic vs. genomic + omics + phenotypes

**Table 7:** Ratio of population accuracies of predicted breeding values for GBLUP, GOBLUP-MI, and GOBLUP-NIR for leave-one-breeding-cycle-out (LBCO) cross-validation

Trait	GBLUPg/gp	GOBLUP-MI			GOBLUP-NIR		
		g/gm	gm/gmp	g/gmp	g/gm	gm/gmp	g/gmp
GY	0,64	0,97	0,62	0,66	0,97	0,61	0,58
PC	0,74	0,92	0,75	0,69	-	-	-
VISC	0,81	1,00	0,81	0,81	0,89	0,80	0,90
BETA	0,79	1,00	0,79	0,79	0,91	0,77	0,87
EXTR	0,81	1,00	0,81	0,80	0,90	0,80	0,73
FILT	0,68	1,00	0,68	0,68	-	-	-
WC	0,86	1,00	0,86	0,86	-	-	-

GY: grain yield; PC: protein content; WV: wort viscosity; BG: β-glucan; EY: extract yield; FS: filtering speed; WC: wort color. GOBLUP_s_g/gm: ratio of accuracies for validation populations with genomic vs. genomic + phenotypes; GOBLUP_s_gm/gmp: ratio of accuracies for validation populations with genomic + omics vs. genomic + omics + phenotypes; GOBLUP_s_g/gmp: ratio of accuracies for validation populations with genomic vs. genomic + omics + phenotypes

## Discussion

The present study used a commercial spring barley breeding population phenotyped for yield, grain protein content, and malting quality traits to investigate the following three research questions. First, we investigated the genetic variation and heritabilities for MIs and NIR wavelengths, and we found a significant proportion of MIs and NIR wavelengths presenting medium to high additive genetic variance and ${\widehat{h}}^{2}$. Second, we assessed the performance of genetic models, including genomic and metabolomic intensities (GOBLUP-MI), or genomic and NIR wavelengths (GOBLUP-NIR), to estimate VCs and heritabilities for all the available traits. We found that GOBLUP-MI and GOBLUP-NIR increase the proportion of genetic variance explained by the model for grain yield, grain protein content, malt extract yield, and β-glucan content. Third, we evaluated the performance of the developed models to predict breeding values, and we generally observed a similar accuracy between GBLUP and GOBLUP-MI, and a worse accuracy for GOBLUP-NIR. Despite this general trend, GOBLUP-MI and GOBLUP-NIR enhanced predictive ability by 4.6 and 2.4% for grain protein in leave-one-breeding-cycle-out and grain yield in fivefold cross-validations, respectively, compared to a baseline GBLUP model; although these differences between models were not statistically significant in a *t*-test (critical *P-value* of 0.01).

### Heritability of metabolomic intensities and NIR wavelengths

The ${h}^{2}$ was investigated using univariate analysis for each of the 30,468 MIs and 141 NIR wavelengths. We observed that ${\widehat{h}}^{2}$ of MIs varied from values < 0.01 to 0.93, and we identified that the heritability of 17,677 MIs (58.02%) were statistically significantly different from zero (Fig. 2). Guo et al. (2020), analyzed a similar barley dataset from the same breeding company, but with MIs obtained from wort (instead of leaf tissue as in our study). In comparison to our study, both studies had a distribution with a high proportion of low ${\widehat{h}}^{2}$ values < 0.1, but in our case, we found a higher proportion of moderate ${\widehat{h}}^{2}$ with values from 0.2 to 0.5 and high ${\widehat{h}}^{2}$ with ${\widehat{h}}^{2}$ > 0.5. In Guo et al. (2020), they found that 35.82% out of 24,018 MIs were significantly different from zero. Despite both studies having defined the significant threshold at 0.01, and having a relevant proportion of significant MIs, the percentage reported by Guo et al. (2020) was lower than in our case. The different statistical power to detect significant ${\widehat{h}}^{2}$ may influence the results, as in our study greater statistical power is expected due to a larger sample size. Also, Guo et al. (2020) included three years of information in the analysis instead of two, which could lead to higher environmental variance and decrease estimates of ${\widehat{h}}^{2}$. The differences between studies might also be explained by having used a different tissue to obtain MIs. Heritability of MIs has also been investigated for other species using different tissues such as fruits and leaves of coffee (Gamboa-Becerra et al. 2019), milk and blood in Holstein cattle (Wittenburg et al., 2013; Aliakbari et al. 2019) and plasma and serum in humans (Frahnow et al. 2017; Hagenbeek et al. 2020), and variable ${\widehat{h}}^{2}$ for MIs (ranging from 0 to > 0.5 for the different tissues) have been found for the different tissues and species. According to our significance threshold estimated at 0.0123, the 41.8% of ${\widehat{h}}^{2}$ of MIs were not significantly different from zero. The large proportion of non-significant ${\widehat{h}}^{2}$ are somehow expected since we used all the available NMR spectra, where some regions may include MI with low or no biological signals (similarly observed by Aliakbari et al. 2019, and Guo et al. 2020). Despite that, using the wide range of MIs may ensure that all potential biological signals can be detected.

The ${\widehat{h}}^{2}$ of NIR wavelengths in our study ranged from < 0.01 to 0.48 (Fig. 3). Rincent et al. (2018) investigated the broad-sense heritability of NIR for leaf and grain tissue of wheat and wood tissue in poplar. These authors reported that the broad-sense heritability was highly variable along the spectrum, with peaks above 60% for both tissues. Similar values of broad-sense heritability of NIR wavelengths from grain tissue in in wheat were found in Robert et al. (2022). The articles of Rincent et al. (2018) and Robert et al. (2022) estimated broad-sense heritability instead of narrow-sense heritability. Our models also allow us to estimate broad-sense heritability by using the total estimated genetic variance (i.e. $d\left(\mathbf{G}\right){\widehat{\sigma }}_{{g}_{j}}^{2}+{\widehat{\sigma }}_{{l}_{j}}^{2}$), and the highest peaks of broad-sense heritability were between 40 and 60%. A possible explanation for the higher values obtained in Rincent et al. (2018) and Robert et al. (2022) may be related to different species involved in the studies. Our result presented on ${\widehat{h}}^{2}$ are novel, as this is the first report on ${\widehat{h}}^{2}$ of MIs for leaf tissue in barley and NIR wavelengths in whole grain after malting.

### Population parameters estimated with genomic and multi-omics models for yield and malting quality traits

Population parameters for GY, PC and MQ traits were estimated with GBLUP and GOBLUP-MI models, and for GY and MQ traits with GOBLUP-NIR. The estimated phenotypic variance (${\widehat{{\varvec{\sigma}}}}_{{\varvec{P}}}^{2}$) was larger for GOBLUP models than for GBLUP. This occurs because the MIs (GOBLUP-MI) and NIR wavelengths (GOBLUP-NIR) capture environmental variance from year-location-trial, that in GBLUP models would be captured by the fixed effects. For GOBLUP-NIR, we observed that the ${\widehat{{\varvec{\sigma}}}}_{{\varvec{P}}}^{2}$ for WV, BG, and EY was higher than the variances of the traits. This issue was also observed, and even more extreme, in Guo et al. (2023) for MQ traits using a GOBLUP model with MIs from wort; possible reasons were thoroughly discussed by these authors and were related to possible wrong model assumptions. According to Christensen et al. (2021) and Guo et al. (2023), possible model deficiencies could be related to: i) wrongly assuming independence and constant heritabilities of omics effects; note that this assumption is needed when using the joint model for prediction of breeding values, but we observed different ${\widehat{h}}^{2}$ for MIs and NIR across the spectra; and ii) assumption of additivity of omics features, which implies that the similarity matrices ${{\varvec{Q}}}_{MI}$ and ${{\varvec{Q}}}_{NIR}$ are matrix cross products of MI and selected principal components of NIR, respectively.

Comparing the GBLUP and GOBLUP-MI, a lower direct genomic variance was observed for GOBLUP-MI for GY and PC. The lower direct genomic variance can be explained due to part of the genetic variance being captured by MIs (${\varvec{u}}$ effect in step 1). A similar and even more extreme trend has been recently observed by Guo et al. (2023) for GOBLUP for MQ traits in barley (Guo et al. 2023). The partition of VCs in GOBLUP-MI revealed that a large proportion of the total variance was captured by MIs for GY and PC (measured by ${\widehat{{\varvec{c}}}}_{{\varvec{m}}}^{2}$ > 0.20). This trend was not observed for MQ traits, where ${\widehat{{\varvec{c}}}}_{{\varvec{m}}}^{2}$ was lower than 0.05 for all traits. Previous reports using GOBLUP with MIs have found higher values of ${\widehat{{\varvec{c}}}}_{{\varvec{m}}}^{2}$ for MQ traits (Guo et al. 2020, 2023). However, an important difference between these studies and ours is that they have used MIs from wort; and therefore, MIs are more directly related to MQ traits. For GY and PC, we see that a substantial metabolome-mediated heritability was obtained with GOBLUP-MI, resulting in higher ${\widehat{h}}^{2}$ than for GBLUP. The differences in ${\widehat{h}}^{2}$ between GBLUP and GOBLUP-MI were smaller for MQ traits. Considering the metabolomic mediated heritability (${h}_{M}^{2}$) estimated for the different traits, a greater potential to include MIs for prediction can be expected for GY and PC than for MQ traits. Additional VC analyses were performed with GOBLUP-MI that tested the performance of using only significant MIs or removing the first 4000 and last 1000 low-signal MIs, but no relevant differences were observed in VCs compared to using all MIs.

For GOBLUP-NIR, a large proportion of total variance was captured by NIR wavelengths for GY, WV, BG, and EY, where ${\widehat{{\varvec{c}}}}_{{\varvec{m}}}^{2}$ was higher than in GOBLUP-MI for the four traits. The ${\widehat{{\varvec{c}}}}_{{\varvec{m}}}^{2}$ in GOBLUP-NIR was especially large for WV (${\widehat{{\varvec{c}}}}_{{\varvec{m}}}^{2}$= 0.81), BG (${\widehat{{\varvec{c}}}}_{{\varvec{m}}}^{2}$= 0.63), and EY (${\widehat{{\varvec{c}}}}_{{\varvec{m}}}^{2}$= 0.85). The large ${\widehat{{\varvec{c}}}}_{{\varvec{m}}}^{2}$ for these traits could be related to a high correlation between NIR wavelengths and grain composition, which is well-established in the literature, and NIR wavelengths are commonly used to predict grain composition and quality in cereals (Dowell et al. 2006; Osborne 2006). In our study, NIR wavelengths have been optimized to predict grain protein content, but still, they may be potentially highly correlated to other quality traits than protein content. Similarly to GOBLUP-MI, the direct genomic variance in GOBLUP-NIR was reduced for WV, BG, ET, and FS, but unexpectedly, an opposite trend was observed for GY. A hypothesis for this opposite trend is that the NIR wavelengths may help to improve the partition of environmental, genetic, and genotype-by-environment interaction effects, resulting in more genetic variance captured by the genomic effect for GY. This could also be true for WV, BG, ET, and FS, but it may be more relevant for traits with higher genotype-by-environment interaction as GY (see VCs in supplementary material 3). Conversely, this could also be related to an upward-biased estimate of direct genomic variance due to wrong model assumptions as described above.

Comparing the ${\widehat{h}}^{2}$ obtained with GOBLUP-NIR and the other models, it was intermediate for GY, highest for BG and EY, and lowest for WV. The GOBLUP-NIR failed to reach convergence of the REML algorithm for FS and WC due to the NIR variance converging towards zero. Several alternatives to get estimates for those traits were utilized, such as trying different starting values for the REML algorithm, using raw or normalized NIR wavelengths instead of the Savitzky-Golay transformation, and using NIR wavelengths of raw grain instead of whole grain after malting, but none of these attempts helped to get VCs for these traits. This could mean that the NIR variance for these traits is not significant and the NIR effect could be excluded from the model, which result in a GBLUP model. Note that principal components were used for NIR wavelengths in GOBLUP-NIR; this strategy is different from the study by Rincent et al. (2018), where they directly used all NIR wavelengths. We believe that no relevant differences in the results should be expected by using all NIR wavelengths or principal components as they explained more than 99% of the variation in NIR wavelengths. However, using the principal components was convenient to facilitate convergence in our study.

### Genomic and multi-omics prediction

The predictive ability (PA), ratio of accuracies according to Legarra and Reverter (2018), and variance dispersion of GEBV (${{\varvec{b}}}_{w,p}$) were evaluated in fivefold and LBCO CVs for GOBLUPs and GBLUP models. The fivefold CV allows us to investigate predictions of models in a favorable scenario as close relatives such as parents, siblings, and half-siblings can be shared between RP and VP, increasing genetic connections between RP and VP. The LBCO CV better reflects the practical conditions in a breeding program, where new lines must be predicted from historical information before the phenotypes are obtained. Thus, the genetic relationships between RP and VP in LBCO are much lower compared to fivefold, and lower PAs are expected (Shao 1993; Kohavi, 1995). On the other hand, different hypotheses can be tested depending on the CV used. In fivefold CV, the accuracy of new lines included in a breeding cycle that were genotyped but not phenotyped can be tested; this investigation is particularly relevant when genotyping is less expensive than phenotyping. The LBCO, alternatively, allows us to test the accuracy of predicting future performance given that lines are genotyped after single seed descent in F_4_.

GBLUP and GOBLUP-MI generally exhibit a similar PA for both CVs, performing better than GOBLUP-NIR for most traits. Despite that, there were some specific cases in each CV where the PA of GOBLUP-MI and GOBLUP-NIR was higher than the PA of GBLUP. In fivefold CV, the GOBLUP-NIR revealed an increase of 2.4% for GY, and in LBCO CV the GOBLUP-MI revealed an increase of 4.6% for PC, although these differences were not statistically significant (critical *P-value* of 0.01). There were also some cases in which the GBLUP outperformed the PA of GOBLUP-MI (GY in both CVs and EY in LBCO CV) and GOBLUP-NIR (WV, BG, and EY in both CVs), but differences were only statistically significant between GBLUP and GOBLUP-NIR in fivefold CV for BG and EY. This trend of lack of improvement in PA with GBLUP-MI is consistent with results by Guo et al. (2023). The reasons for the lower PA using the GOBLUPs models could be related to deficiencies in model assumptions, as discussed in the previous section.

The ratios of population accuracies were analyzed for fivefold and LBCO CVs. We observed a moderate to high improvement in including phenotypic information in VP in fivefold and LBCO CVs, respectively. This is expected as a higher response for including new information is generally obtained in more restrictive scenarios. No relevant improvement of including MIs or NIR wavelengths was observed in any of the CVs and models; these results were consistent with the observed in the analysis of PA. In contrast, a substantial improvement for including MIs was observed using the ratios of accuracies by Guo et al. (2023). Importantly, Guo et al. (2023) used MIs from wort instead of leaf tissue to assess MQ traits, which could be the main explanation for the differences observed. Comparing our study and Guo et al. (2023), we observed that the sampling stage and tissue was relevant for MQ traits, with the highest benefit observed for MI sampled in wort. Nevertheless, sampling MI in the wort may not represent an optimal strategy, as this requires all the malting steps incurring extra cost. Further studies exploring alternative sampling stages and tissues to define what is the best sampling strategy either to obtain MI or NIR wavelengths for the different traits are warranted. No variance inflation (${b}_{w,p}$) was found for any of the traits in fivefold CV; however, moderate over-dispersion was found for GY in LBCO CV. A possible explanation for this may be related to the fact that in LBCO CV each breeding cycle is assessed in a different year. This could result in an unbalanced scenario to predict traits with large genotype-by-environment interactions as lines assessed in one year are used to predict outcomes for a different breeding cycle tested in a different year, which could lead to issues of variance inflation (Raffo and Jensen 2023). Additional analyses were performed with GOBLUP-MI by testing the predictive performance of using only significant MIs or removing the first 4000 and last 1000 low-signal MIs, but a similar or a lower performance were observed compared to using all MIs. Further studies are warranted to explore alternative sampling strategies for identifying the optimal stage and tissue and to investigate the impact of violating assumptions in GOBLUP models.

## Conclusions

In this study, we used a commercial barley breeding population to investigate the viability of including metabolomic intensities sampled from early flag leaves, and near-infrared wavelengths, sampled from whole grain after malting, for genomic evaluations of yield and malting quality traits. First, we concluded that a significant proportion of metabolomic intensities and near-infrared wavelengths had medium to high additive genetic variance and heritabilities (${\widehat{h}}^{2}$) and can, therefore, be potentially useful for genetic evaluations. Second, we concluded that multi-omics models including genomic and metabolomics (GOBLUP-MI), or genomic and NIR wavelengths (GOBLUP-NIR), increased the proportion of genetic variance explained by the models for grain yield, grain protein content, malt extract yield, and β-glucan content, compared to a purely genomic model (GBLUP). Third, we assessed genomic and multi-omics models for prediction of breeding values, and we concluded that GBLUP and GOBLUP-MI had a similar prediction accuracy, and performed better than GOBLUP-NIR for most traits. Despite that, the GOBLUP-MI and GOBLUP-NIR models slightly improved accuracy of predicting breeding values compared to the GBLUP for some specific traits, but differences were not statistically significant in a *t*-test. The different performance of GOBLUPs models across traits might be related to different aspects specific to each trait (e.g. genetic architecture, influence of genotype-by-environment interactions), and the sampling strategy to obtain metabolomic intensities or near-infrared wavelengths. For malting quality traits, sampling leaf tissue revealed worse performance for GOBLUP-MI compared to previous research that had sampled wort. The lack of advantage confered in our case is likely attributed to the sampling strategy and not to the method utilized.

## Supplementary Information

Below is the link to the electronic supplementary material.

Supplementary file1 (PDF 1714 KB)

Supplementary file2 (PDF 320 KB)

Supplementary file3 (XLSX 43 KB)

## Data Availability

The datasets analyzed during the current study are available in the Harvard dataverse repository at the following link: https://dataverse.harvard.edu/dataset.xhtml?persistentId=doi:10.7910/DVN/K3OFFI
