# Supplementary material for: Genomic prediction for yield and malting traits in barley using metabolomic and near-infrared spectra
Source: Theor Appl Genet. 2025 Jan 9;138(1):24. doi: 10.1007/s00122-024-04806-7 (PMC11717810; doi:10.1007/s00122-024-04806-7)
Supplement: Supplementary file 1 — Supplementary file1 (PDF 1714 KB) [file 122_2024_4806_MOESM1_ESM.pdf]

Supplementary material 1

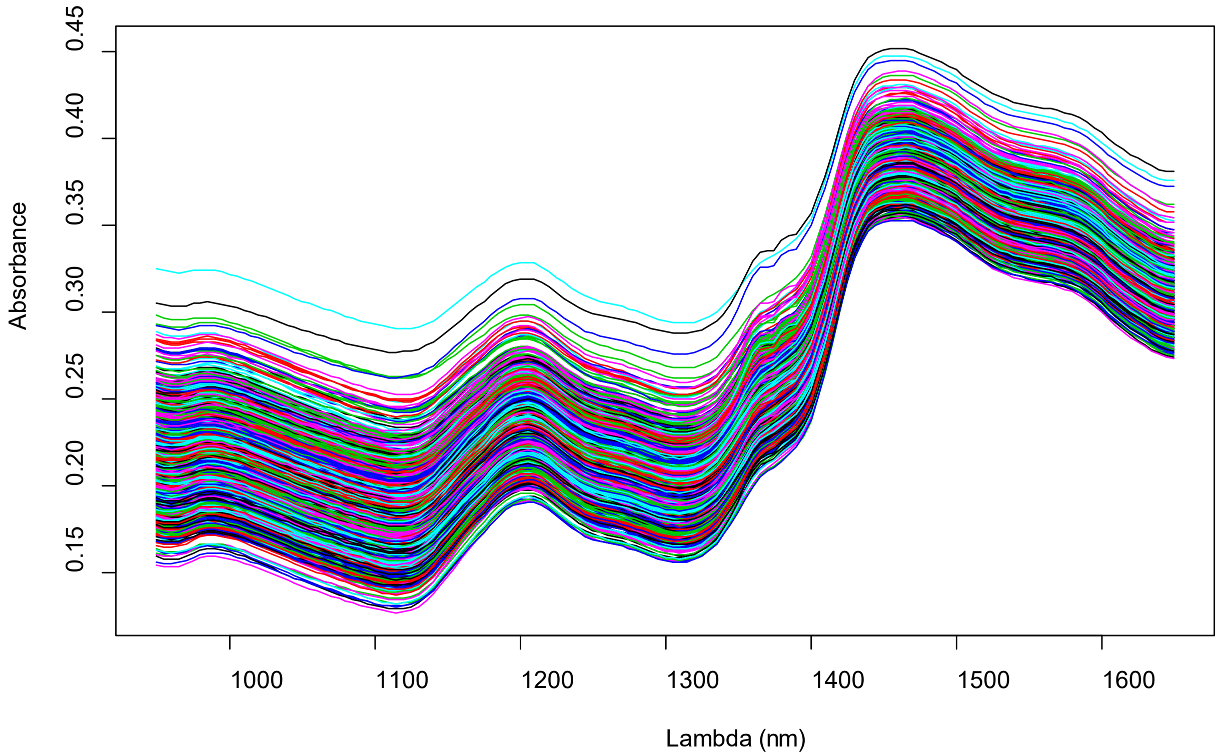

Figure S1. Change in absorbance from 950 to 1650 nm for raw NIR wavelengths (lines with different colors) for whole barley grain after malting.

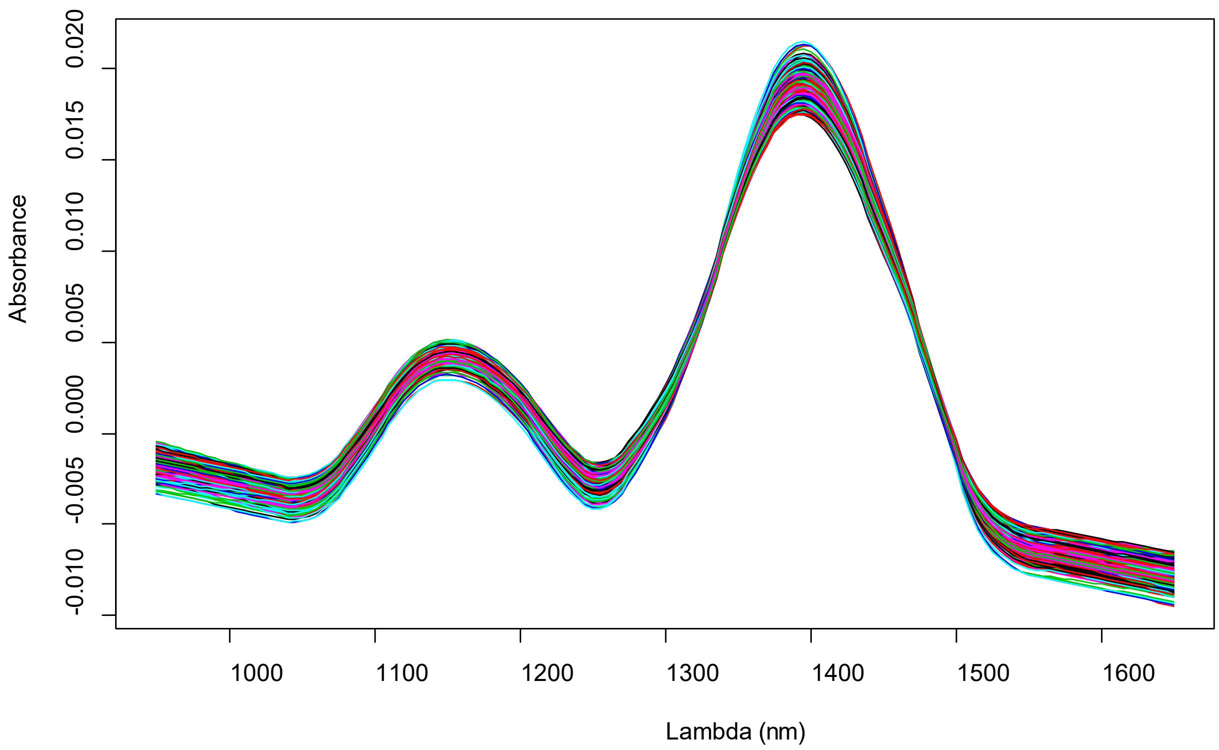

Figure S2. Change in absorbance from 950 to 1650 nm for NIR wavelengths after Savitzky-Golay transformation (lines with different colors) for whole barley grain after malting.
