# Supplementary material for: Genomic prediction for yield and malting traits in barley using metabolomic and near-infrared spectra
Source: Theor Appl Genet. 2025 Jan 9;138(1):24. doi: 10.1007/s00122-024-04806-7 (PMC11717810; doi:10.1007/s00122-024-04806-7)
Supplement: Supplementary file 2 — Supplementary file2 (PDF 320 KB) [file 122_2024_4806_MOESM2_ESM.pdf]

Supplementary material 2

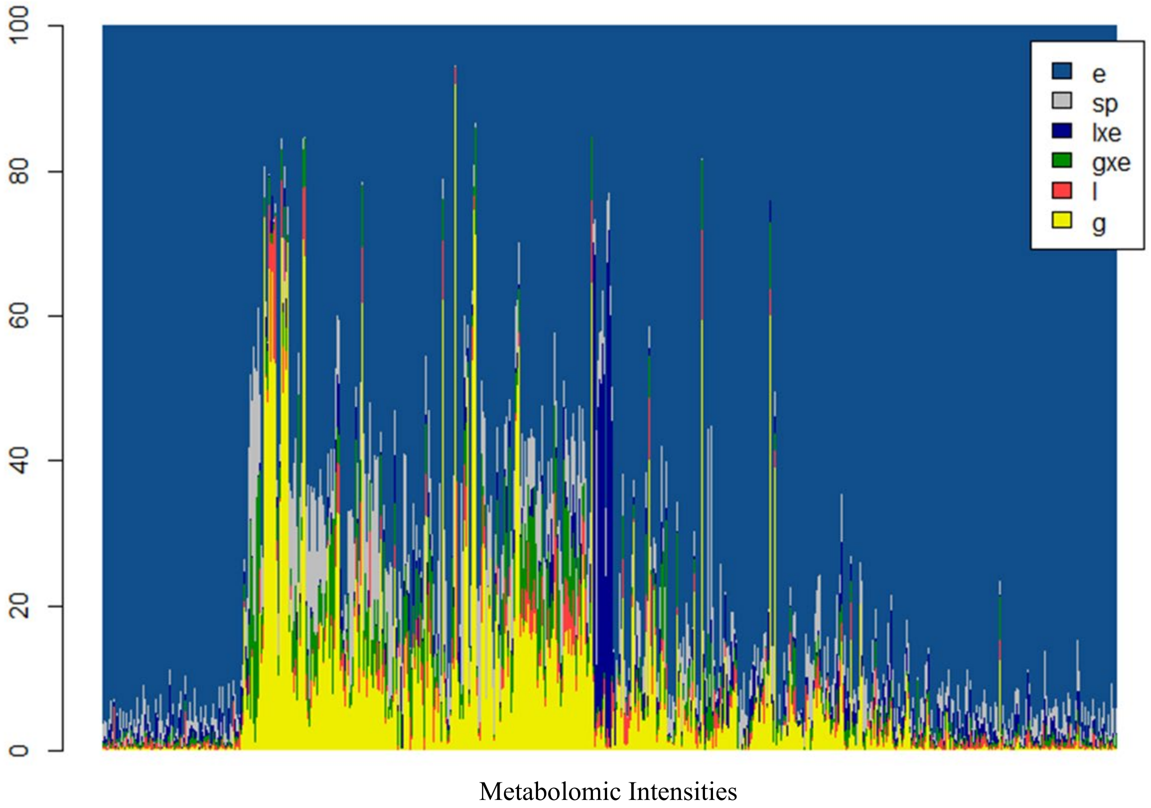

Figure S3. Relative proportion (%) of variance components for metabolomics intensities (MIs); g: additive genomic variance; l: line variance; gxe: genomic additive-by-environment interaction variance; lxe: line-by-environment interaction variance; sp: spatial variance; e: residual variance.

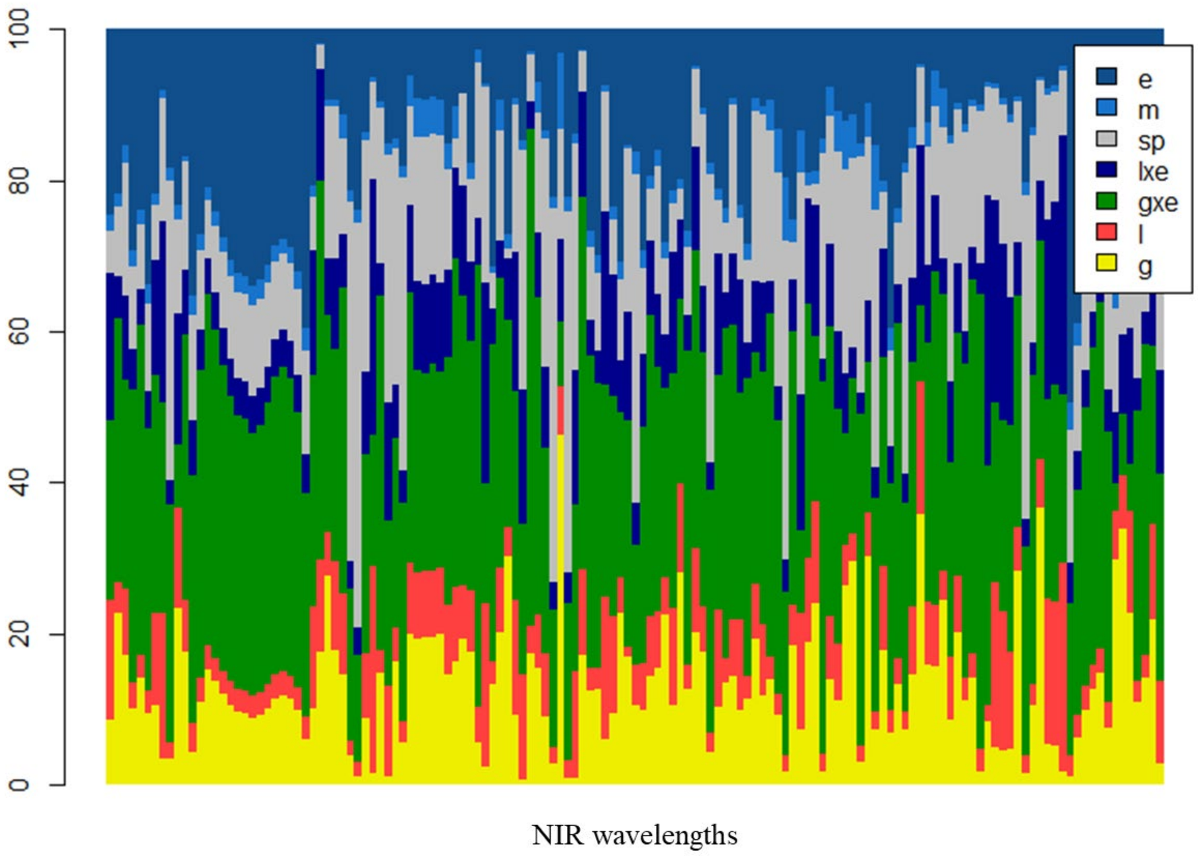

Figure S4. Relative proportion (%) of variance components for NIR wavelengths; g: additive genomic variance; l: line variance; gxe: genomic additive-by-environment interaction variance; lxe: line-by-environment interaction variance; sp: spatial variance; m: malting group variance; e: residual variance.
